# ZnO Nanowire-Based Corona Discharge Devices Operated Under Hundreds of Volts

**DOI:** 10.1186/s11671-015-1217-4

**Published:** 2016-02-16

**Authors:** Wenming Yang, Rong Zhu, Xianli Zong

**Affiliations:** School of Mechanical Engineering, University of Science and Technology Beijing, Beijing, 100083 China; State Key Laboratory of Precision Measurement Technology and Instruments, Department of Precision Instruments, Tsinghua University, Beijing, 100084 China

**Keywords:** ZnO, Nanowires, Corona discharge, Corona inception voltage, Electric field enhancement

## Abstract

Minimizing the voltage of corona discharges, especially when using nanomaterials, has been of great interest in the past decade or so. In this paper, we report a new corona discharge device by using ZnO nanowires operated in atmospheric air to realize continuous corona discharge excited by hundreds of volts. ZnO nanowires were synthesized on microelectrodes using electric-field-assisted wet chemical method, and a thin tungsten film was deposited on the microchip to enhance discharging performance. The testing results showed that the corona inception voltages were minimized greatly by using nanowires compared to conventional dischargers as a result of the local field enhancement of nanowires. The corona could be continuously generated and self-sustaining. It was proved that the law of corona inception voltage obeyed the conventional Peek’s breakdown criterion. An optimal thickness of tungsten film coated over ZnO nanowires was figured out to obtain the lowest corona inception voltage. The ion concentration of the nanowire-based discharger attained 10^17^/m^3^ orders of magnitude, which is practicable for most discharging applications.

## Background

A corona discharge arises from an ionization and partial breakdown of surrounding atmospheric gases at a highly curved electrode within a strong electric field. It can bring about the stationary generation of ions with relatively low electric current and has been used as an ionizer in various areas such as aerosol measurements [1], degradation of organic compounds [2], preparation of some types of nanomaterials [3], mass spectrometers [4], and micromotors [5]. Most of these discharge schemes are achieved by using needle-to-plane electrode geometries, where needles with smaller diameters can generate a greater local electric field enhancement when other parameters are fixed. Accordingly, it is of great interest to develop a miniaturized corona ionizer whose corona inception voltage could be reduced while the ion concentration could be maintained at an available level with the aid of minimizing needles. For instance, ionization gas sensors realized their on-chip operation due to the contribution of various nanostructures including nanotubes [6], silicon nanowires [7], gold nanowires [8], ZnO nanowires [9], and other nanostructures [10, 11]. These discharges in gas sensors do not need to be maintained continuously, but only the breakdown voltages are detected to discriminate ambient gases.

Recent studies on the ionization of atmospheric air have paid their attentions to continuous corona discharges with microscale or nanoscale needles which permit the reduction in physical size and cost of the corona ionizers. Eifert et al. [12] used the nanostructured edges of gold and aluminum foils as electrodes with 5-mm inter-electrode spacing for corona discharges. The lowest corona inception voltage reached 1.2 kV in ambient air. Hsu et al. [13] fabricated a cantilever-like corona electrode with a tip radius of about 500 nm and electrode gaps of 1–6 mm. The smallest corona onset voltages were in the range of 2–2.5 kV. Chua et al. [14] developed a micromachined corona ionizer with a point-to-grid geometry and millimeter electrode gaps. The corona inception voltages were in the range from 1.4 to 2 kV. Park et al. [15] designed a chip-type unipolar discharger with the inter-electrode gap of 165 μm. The corresponding corona inception voltage was 1.5 kV, and the estimated ion concentration was in the range from 6.5 × 10^12^/m^3^ to 4.3 × 10^13^/m^3^. Bo et al. developed nanomaterial-based corona discharges using suspended carbon nanotubes [16] and graphene sheets grown on metallic wires [17] to realize a continuous corona discharge at a corona inception voltage of about 0.1 and 3.2 kV, respectively. The maximum surface current density of the corona discharge they obtained using the suspended carbon nanotubes was 0.095 A/m^2^ at the voltage of 0.9 kV.

Nanowires are excellent candidates to enhance local electric fields for corona discharges, and among them, ZnO nanowires have the advantages of efficient excitonic emission [18, 19] and high temperature resistance, which could efficiently prevent the electrodes from damage due to ion bombardment and unsteady ion currents. The employment of ZnO nanowires to carry out a continuous corona discharge was demonstrated by Yang et al. [20]. Later, Park et al. [21] reported a ZnO nanowire charger with an interstitial gap of 0.9 mm between electrodes, whose corona starting voltage was 1.6 kV and the generated ion concentrations were 6.7 × 10^8^/cm^3^–4.3 × 10^9^/cm^3^ at the applied voltages of 2–3.1 kV.

In this paper, we presented a comprehensive design and analysis on the continuous corona dischargers using ZnO nanowires, which were synthesized on microelectrodes using an electric-field-assisted wet chemical method and coated by a thin tungsten film to enhance their conductivity and discharging performance. Electric field enhancements of ZnO nanowires were studied, and the corona inception voltages were experimentally tested. The performances of the corona discharge devices using ZnO nanowires demonstrate their promising prospects for versatile applications.

## Methods

### Growth of ZnO Nanowire on Microelectrodes

ZnO nanowires as the anodes of ionizers were grown on microelectrodes using an electric-field-assisted wet chemical method, which is with low temperature, simplicity, low cost, capable of easy scale-up compared with other methods, and easy to incorporate with micromachined electrode structures [22, 23]. The electric field was used to control the growth position and growth direction of nanowires on the electrodes by applying a tailor-designed electric field. The shape of the microelectrodes was designed as a coplanar comb-finger pair which was favorable for the growth of nanowires [24].

**Fig. 1:**
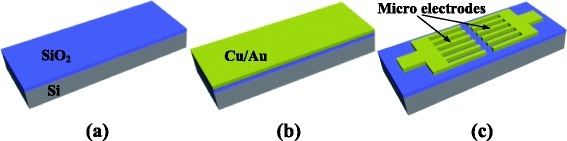
Schematic diagram of the fabrication process for microcomb-finger electrodes. **a** Si wafer with ~500 nm of deposited SiO_2_. **b** Wafer after deposition of ~100 nm of Cu/Au. **c** Final wafer after patterning of Cu/Au. The images are not to scale

The fabrication of the microcomb-finger electrodes is shown in Fig. 1, a N-doped silicon wafer with a low resistivity of 0.008~0.02 Ω cm was firstly prepared, and then, a thin layer of SiO_2_ with a thickness of 500 nm was deposited on the silicon wafer by thermal oxidation. Afterwards, a 100-nm Cr/Au film was sputter-deposited and patterned using photolithography onto the SiO_2_ layer, and hereafter, the electrode pairs taking the form of comb-like structure were formed. Additionally, the connection between the electrodes with outside pins was constructed using wire bonding.

**Fig. 2:**
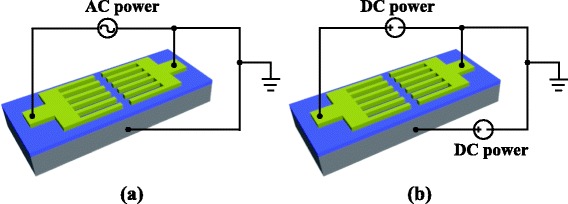
Electric field control growing of ZnO nanowires. **a** AC controlled process. **b** DC controlled process

Using the fabricated sample above, ZnO nanowires were grown using a wet chemical method with the assistance of an applied electric field. The whole sample was immersed in an equal molar aqueous solution (0.015 M) of zinc nitrate hydrate (Zn(NO_3_)_2_·6H_2_O) and hexamethylenetetramine (C_6_H_12_N_4_), which was heated at 75 °C. The nanowire growth can be controlled by AC or DC electric field. During an AC controlled process, a sine wave voltage (1 MHz, 4–5 Vpp) was applied between the top electrode pair while the bottom N-doped silicon substrate was grounded. In contrast, during a DC controlled process, a DC voltage between the top electrode pair (−0.35 V) and another DC voltage between the bottom substrate and one of the top electrodes (−0.1 V) were applied onto the electrodes, as shown in Fig. 2. After 3.5 h of the growing process, the sample was taken out from the solution, rinsed with deionized water using ultrasonic cleaning, and dried in air.

**Fig. 3:**
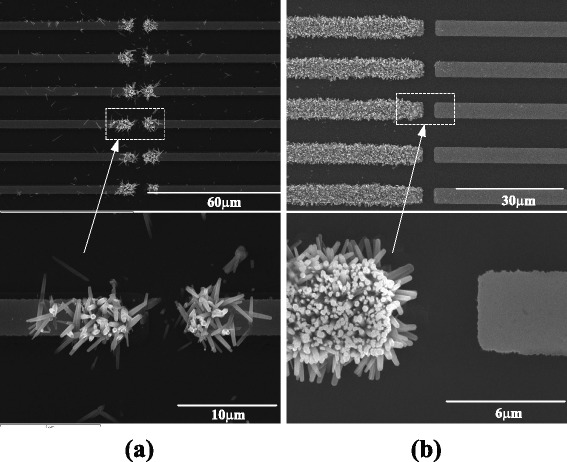
SEM images of ZnO nanowires grown on microelectrodes. **a** The growth of nanowires was controlled by applying AC electric field. **b** The growth of nanowires was controlled by applying DC electric field

Figure 3 shows the SEM images (top view) of the ZnO nanowires grown on microelectrodes by AC and DC electric field controls, respectively. ZnO nanowires were grown at the opposite ends of the electrode pair and predominantly along the lateral direction by AC control and were grown only on one of the electrodes (cathode) and predominantly along the vertical direction by the DC control. The grown nanowires with diameters of 100–600 nm and lengths of 1–3 μm have hexagonal cross sections. The average number densities of the nanowires by DC and AC controls were about 4.8 × 10^11^/m^2^–7.6 × 10^11^/m^2^ and 5.4 × 10^10^/m^2^–1.4 × 10^11^/m^2^, respectively. EDX results had shown the atomic ratio of zinc to oxygen was near stoichiometric composition (1:1) which demonstrated the growth of ZnO. TEM tests and SAED patterns indicated the single crystal structure of ZnO nanowires and their <0001> growth direction. After the growth of ZnO nanowires, a thin metal film was deposited on the wafer by sputtering (JB-2B sputtering machine) to enhance the conductivities and discharging performance of the nanowires.

### Assembly of Devices

**Fig. 4:**
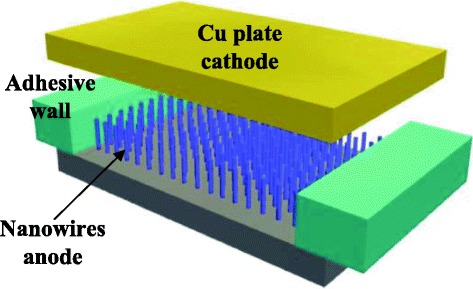
Schematic view of a corona discharger using nanowires

After the chip fabrication, a silicon transfer film adhesive with a thickness of 50 μm (ARclad® IS-7876, adhesive research, USA) was used as the adhesive wall to assemble a thin copper plate or a piece of indium tin oxide (ITO) glass on the prepared wafer to form a discharger as shown in Fig. 4. The microelectrode covered with nanowires was served as the anode of the discharger and the copper plate or ITO glass as the cathode. The gap distance between the anode and cathode was about 48 μm. This geometry of the discharger approaches the “standard” point-to-plane geometry of the corona discharge where the ratio of gap distance to the radius of the point should equal to 160 [25].

### Experimental Method

**Fig. 5:**
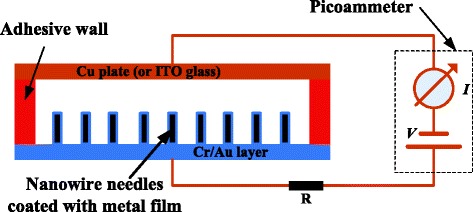
Diagram of experimental setup

Experiments were conducted to study the performances of the developed dischargers. The *I*-*V* characteristics and the breakdown voltages were measured using a Keithley 237 picoammeter, the experimental setup of which is depicted in Fig. 5. A limiting resistor *R* was connected into the circuit to prevent heavy current when breakdown happened. The applied voltage was increased in discrete steps with an increment *V*_s_, which was decreased when it approached the breakdown threshold. Time duration in tens of seconds was maintained at each step. For each applied voltage, the *I*-*V* data were recorded and the discharge currents were time-averaged by using a developed LabView program.

## Results and Discussion

### Electric Field Enhancement of ZnO Nanowires

**Fig. 6:**
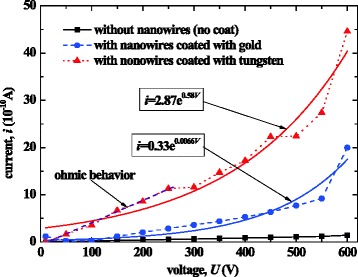
*I*-*V* characteristics of the discharger using nanowires coated with gold or tungsten film and the comparison with the discharger without nanowires

Two kinds of metallic film were used to deposit on the surface of the wafer: one is gold and the other is tungsten. Figure 6 compares the tested *I*-*V* characteristics of the dischargers with nanowires (grown by AC control) coated by gold or tungsten film (with the same thickness of 80 nm, nearly the same number density 1.4 × 10^11^/m^2^ of nanowires) compared with the discharger without nanowires. The coated tungsten film enhanced the conductivity almost two times larger than that coated with gold.

According to the traditional gas discharge theory [26], in the regime before the point of the breakdown, ionization occurs at first via random events and then predominantly through electron impact ionization. The ionization current *i* in this regime can be expressed as

1 $$ i=\frac{i_0{{\mathrm{e}}^{\alpha}}^{d_0}}{1-\gamma \left({\mathrm{e}}^{\alpha {d}_0}-1\right)}, $$

where *i*_0_ is the current due to electrons liberated from the cathode by external radiation, *α* is known as Townsend’s first ionization coefficient, *γ* is known as Townsend’s secondary ionization coefficient, and *d*_0_ is the electrode gap. Both *α* and *γ* are strong functions of the reduced field (*E*/*p*, where *E* is electric field and *p* is pressure). As seen in Fig. 6, the response of current as a function of the applied potential showed an exponential shape, suggesting that a Townsend discharge was the dominant phenomenon. Numerous space charges produced in the Townsend discharge should enter the electrodes to form the discharge current, among which electrons and other negative ions gathering in the gap would be absorbed into the anode. However, the entrance would be easier and more quick for the tungsten-coated anode owing to its lower work function. Therefore, the Townsend discharge current of the discharger with nanowires coated by tungsten was larger than that with the gold film.

In geometrical configurations resembling a parallel-plate capacitor, the macroscopic electric field *E*_M_ is uniform in the space between electrodes and can be defined by *E*_M_ = *U/L,* where *U* is the voltage applied across a gap of thickness *L*. Nanowires placed on either electrode can generate very high nonlinear local electric fields *E* near the tips, owing to their high aspect ratio. The enhancement effect is represented by a field enhancement factor defined as

2 $$ \beta =\frac{E}{E_{\mathrm{M}}} $$

The *I*-*V* characteristic of the discharger with tungsten-coated nanowires exhibits a near ohmic behavior in the region with lower voltage as shown in Fig. 6. The discharge current density in this region can be denoted by

3 $$ J={\sigma}_{\mathrm{Gas}}E, $$

where *σ*_Gas_ is the gas conductivity. If *σ*_Gas_ is assumed to be constant at fixed ambient conditions (temperature, humidity, and radiation), *β* can be estimated by comparing the measured discharge currents of the discharger with tungsten-coated nanowires to that without nanowires, namely

4 $$ \beta \approx \frac{i}{i_{\mathrm{M}}} $$

where *i*_M_ is the current of discharger without nanowires.

**Fig. 7:**
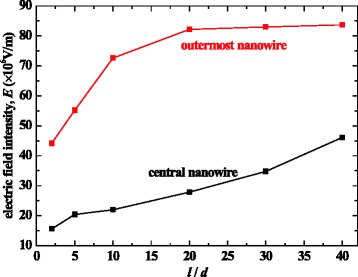
Electric field intensity as a function of ratio *l*/*d* at the tip tops of the central and the outermost nanowire (*U* = 250 V, *d* = 350 nm, and *h* = 3μm)

The field enhancement factor can also be estimated by the numerical calculation of the electric field. The electric field intensities at the tip tops of the central and the outermost nanowire versus the ratio *l*/$d$ were calculated using Ansys software when $U$ = 250 V, $d$ = 350 nm, and ℎ = 3 μm, where *l* is the distance between nanowires and *d* and *h* are diameter and length of nanowires, respectively, the results of which are shown in Fig. 7. It is shown that the electric field at the tip of the central nanowire was lower than that for the outermost nanowire. For the outermost nanowire when *l*/$d$ > 20 (this condition was met for most of the AC-grown and some DC-grown ZnO nanowires located at the ends of microelectrodes), nearly constant field intensity was found, whose value approaches that in the case where there is only one nanowire on the electrode. These results demonstrate that the local electric fields at the tip tops of the outermost nanowires are not altered by the interaction between the tips which are further than a few micrometers apart. In this case, the “hemisphere on post” model [27] can be employed on the outermost nanowires to estimate *β* based on the length *h* and radius *r* of nanowires:

5 $$ \beta =1.2{\left(2.15+h/r\right)}^{0.90}. $$

Table 1 lists the values of *β* calculated by Eq. (4) from experiments, Eq. (2) from the numerical simulation of the electric field, and Eq. (5) from the “hemisphere on post” model. There is a large difference between the experimental *β* and those derived from simulation and the model, which is mainly induced by the assumption of constant gas conductivity. The gas conductivity depends on charge density and electrical mobility of the charges. When the corona happens, there is a higher level of ion concentration distribution near the corona discharge region. The resultant increase of gas conductivity results in a larger value of *β* by the substitution of *E*/*E*_M_ with *i/i*_M_. Another factor for the difference may be attributed to the ionization contributed by multiple nanowires.

**Table 1:** Values of field enhancement factor *β* calculated by different methods

Method	Experimental result	Numerical simulation	“hemisphere on post” model
*β*	21.7	16.7	17.2

Further increasing voltage applied on the discharger with the tungsten film could yield a continuous corona discharge, while that with the gold film could only achieve a transient spark. As a result, tungsten was selected as the coating film.

### Corona Discharge Phenomenon

**Fig. 8:**
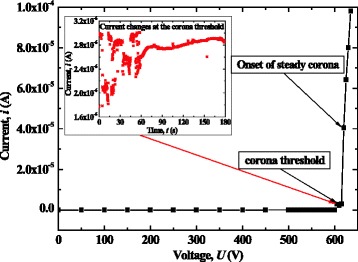
The typical *I*-*V* characteristics of the dischargers using nanowires coated with tungsten film

**Fig. 9:**
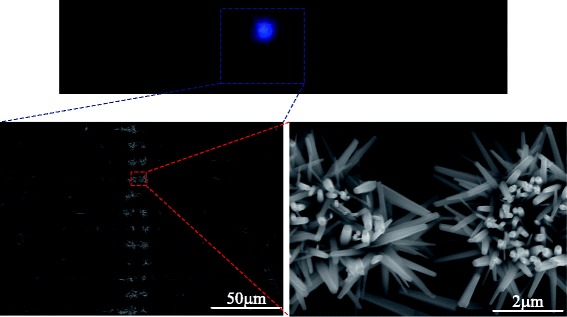
Image of the bluish luminescence of the corona discharge and its location on the chip

A continuous and stable corona discharge could be obtained in atmosphere using the discharger with tungsten-coated nanowires. This nanowire-based corona discharge could be initiated and operated at a relatively lower voltage due to downsizing of the electrode tip by using nanowires. Figure 8 shows tested currents versus applied voltages for a developed discharger. When the applied voltage was increased to the corona threshold, a breakdown happened (a dramatic change was found for the current). At this time, the current was in the order of microampere, unsteady, and fluctuated dramatically as shown in the inset of Fig. 8. If we keep increasing the voltage so as to be slightly larger than the corona threshold, the corona discharge became steady. The voltage corresponding to the onset of steady corona is denoted as the “corona inception voltage.” After the corona inception, a steady bluish luminescence was observed near the tips of the nanowires, accompanied by a hissing noise. Figure 9 shows the continuously bluish luminescence and its location on the chip. Obviously, a continuous corona discharge took place at the tips of the nanowires. Further increasing the applied voltage obtained a linear *I*-*V* characteristic. The dischargers with nanowires grown by AC and DC control had a similar discharge process.

### Corona Inception Voltage

**Fig. 10:**
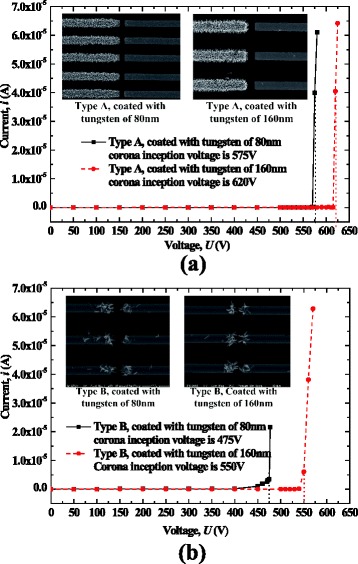
Loading currents of nanowire-based dischargers with respect to the applied voltage. **a** “type A” dischargers. **b** “type B” dischargers

Figure 10 shows the currents of the dischargers using AC-grown and DC-grown nanowires with different thicknesses of tungsten film (80 or 160 nm), where “type A” and “type B” refer to the dischargers using DC-grown nanowires and AC-grown nanowires, respectively. The corresponding corona inception voltages of these dischargers were all lower than 650 V, which are greatly less than most other reported dischargers (about several kilovolts). Further comparison of the corona inception voltages of type A to type B indicates that type B using nanowires grown by AC control had lower corona inception voltages than type A using nanowires grown by DC control. The reason is that the nanowires grown by AC control were more scattered and had a lower number density than those by DC control. The enhancement of the electric field for the nanowires grown in the lateral direction is greater than that for the nanowires grown vertically.

Peek [28] has arrived at a well-known semi-empirical equation describing the electric field strength to initiate a visual corona, which is known as Peek’s breakdown criterion. A modified version of this criterion considering the surface roughness is given as follows:

6 $$ {E}_{\mathrm{C}}=Af\delta \left(1+B\sqrt{\frac{1}{\delta r}}\right) $$

where *r* is the tip radii of curvature of the electrode where the ionization takes place, *δ* is the relative air density, and *f* = 0.6 for most applications. *A* and *B* are coefficients empirically derived, which are commonly given as approximately 30 kV/cm and 0.3 cm^1/2^, respectively. Although these two coefficients are only available for three specific electrode configurations including wire-in-cylinder, parallel wires, and spheres of identical radius, the needle-to-plane configuration may be approximated with similar configurations using the same characteristic length [14]. For nanowires with radii of 180 nm and coated tungsten film of 80 and 160 nm, the critical electric field strength given by Eq. (6) is 116.9 and 107.7 V/m, respectively. Moreover, the critical electric field strength can also be estimated by the maximum electric field strength at the tips of nanowires, which may be calculated using the numerical method based on the experimental results of corona inception voltage and the actual maximum distance between nanowires. Table 2 shows the results obtained by this method and by Eq. (6) for the four cases shown in Fig. 10. The close proximity of the critical electric field strength calculated by these two methods confirms that the corona discharge using ZnO nanowires is accordant with Peek’s breakdown criterion.

**Table 2:** Critical electric field strength calculated by Eq. (6) and the numerical method (the unit in the table is V/m)

Discharger	Type A, tungsten film 80 nm	Type B tungsten film 80 nm	Type A tungsten film 160 nm	Type B tungsten film 160 nm
Calculated by Eq. (6)	116.9	116.9	107.7	107.7
Calculated numerically	109.0	110.2	96.7	98.8

**Fig. 11:**
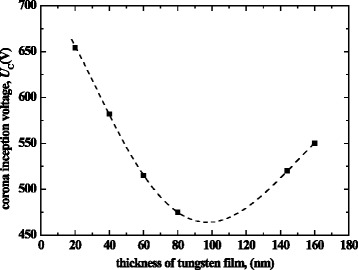
Corona inception voltages as a function of thickness of tungsten film for nanowire-based dischargers

The thickness of the tungsten film coated on nanowires is another factor influencing corona inception voltage. Figure 11 shows the variation of corona inception voltage with respect to the thickness of tungsten film for type B dischargers. Note that there is an optimal thickness to obtain the lowest corona inception voltage. This is because too thin tungsten films have limited improving effectiveness on the ZnO nanowire conductivity, but the dull effect on the tips of nanowires will be prominent if the tungsten film is too thick.

### Ion Concentration Induced by Corona Discharge

**Fig. 12:**
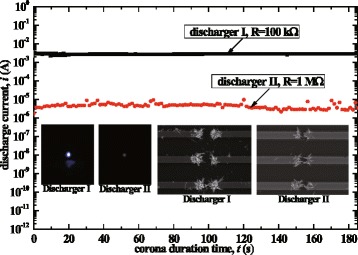
Discharge currents of the nanowire dischargers using different current limit resistors and images of the steady bluish luminescence when the steady corona occurred

Connecting a limiting resistor into the measuring circuit could modulate the corona discharge current such that the resistor changed the voltage to sustain the corona duration after it was ignited, the results of which for type B dischargers using different resistors *R* are shown in Fig. 12. The corona currents were 10^−3^ and 10^−6^ A orders of magnitude corresponding to *R* = 100 kΩ and 1 MΩ, respectively, which also corresponded to different brightness of the corona luminescence as shown in the inserts of Fig. 12.

The ion concentration (*N*_i_) produced in the corona discharge was estimated using the following expression [29]:

7 $$ {N}_{\mathrm{i}}=\frac{I_{\mathrm{c}}}{e{Z}_{\mathrm{i}}EA}, $$

where *I*_c_ is the experimentally tested corona currents shown in Fig. 12, *e* is the elementary charge value (1.6 × 10^−19^ C), *Z*_i_ refers to the mobility of positive air ions (1.4 × 10^−4^ m^2^/Vs), *E* refers to the mean value of the applied electric field, and *A* is the surface area of the electrodes. The surface area of the electrode for the type B discharger was estimated to be about 2.5 × 10^−5^ m^2^. According to Eq. (7), the ion concentrations of the two dischargers shown in Fig. 12 were estimated to be about 3.8 × 10^17^/m^3^ (surface current density 40 A/m^2^, which are larger than other reported dischargers, discharger I) and 4.0 × 10^13^/m^3^ (surface current density 0.24 A/m^2^, discharger II), respectively.

## Conclusions

Continuously self-sustained corona discharges using ZnO nanowires as anode tips of dischargers are realized. The corona inception voltage is greatly reduced to hundreds of volts in virtue of the local field enhancement by nanowires as electrodes. It is proved that the law of corona inception voltage obeys Peek’s breakdown criterion. An optimal thickness of tungsten film coated on ZnO nanowires is figured out to obtain the lowest corona inception voltage. The ion concentration of the developed corona discharger using nanowires attains 10^17^/m^3^ orders of magnitude, which is sufficient for most applications.

## References

[1] Kulkarni P, Baron PA, Willeke K (2011). Aerosol measurement, principles, techniques, and applications.

[2] Klett C, Touchard S, Vega-Gonzalez A, Redolfi M, Bonnin X, Hassouni K, Duten X (2012). Experimental and modeling study of the oxidation of acetaldehyde in an atmospheric-pressure pulsed corona discharge. Plasma Sour Sci Technol.

[3] Brock JR, Texas PL (1991). Formation of carbon fibers in corona discharges. Appl Phys Lett.

[4] Skalny JD, Orszagh J, Mason NJ, Rees JA (2008). Mass spectrometric study of negative ions extracted from point to plane negative corona discharge in ambient air at atmospheric pressure. Int J Mass Spectrosc.

[5] Leea S, Kim D, Michael DB, Ling FF (2005). A micro corona motor. Sens Actuators A.

[6] Modi A, Lass NK, Wie EB, Ajayan PM (2003). Miniaturized gas ionization sensors using carbon nanotubes. Nature.

[7] Sadeghian RB, Islam MS (2011). Ultralow-voltage field-ionization discharge on whiskered silicon nanowires for gas-sensing applications. Nat Mater.

[8] Sadeghian RB, Kahrizi M (2007). A novel miniature gas ionization sensor based on freestanding gold nanowires. Sens Actuators A.

[9] Liao L, Lu HB, Shuai M, Li JC, Liu YL, Liu C, Shen ZX, Yu T (2008). A novel gas sensor based on field ionization from ZnO nanowires: moderate working voltage and high stability. Nanotechnology.

[10] Go DB, Fisher TS, Garimella SV, Bahadur V (2009). Planar microscale ionization devices in atmospheric air with diamond-based electrodes. Plasma Sour Sci Technol.

[11] Liu H, Yadian B, Liu Q, Gan CL, Huang YZ (2013). A hybrid nanostructure array for gas sensing with ultralow field ionization voltage. Nanotechnology.

[12] Eifert A, Baier T, Hardt S (2013). Small onset voltages in negative corona discharges using the edges of gold and aluminum foils as nano-structured electrodes. Appl Phys Lett.

[13] Hsu CP, Jewell-Larsen NE, Krichtafovitch IA, Mamishev AV (2009). Miniaturization of electrostatic fluid accelerators. J Microelectromech Syst.

[14] Chua B, Wexler AS, Tien NC, Niemeier DA, Holmen BA (2008). Design, fabrication, and testing of a microfabricated corona ionizer. J Microelectromech Syst.

[15] Park DH, Kim YH, Lee SG, Kim C, Hwang J, Kim YJ (2010). Development and performance test of a micromachined unipolar charger for measurements of submicron aerosol particles having a log-normal size distribution. J Aerosol Sci.

[16] Bo Z, Yu K, Lu G, Mao S, Chen J, Fan F (2010). Nanoscale discharge electrode for minimizing ozone emission from indoor corona devices. Environ Sci Technol.

[17] Bo Z, Yu K, Lu G, Cui S, Mao S, Chen J (2011). Vertically oriented graphene sheets grown on metallic wires for greener corona discharges: lower power consumption and minimized ozone emission. J Energy Environ Sci.

[18] Cui JB (2012). Zinc oxide nanowires. Mater Charact.

[19] Fang Y, Wong KM, Lei Y (2012). Synthesis and field emission properties of different ZnO nanostructure arrays. Nanoscale Res Lett.

[20] Yang WM, Zhu R, Zong XL. Continuous corona discharge using nanowires. The Fourth International Conference on Manipulation, Manufacturing and Measurement on the Nanoscale; 27–31 Oct 2014. p 143–7.

[21] Park CW, Lee S, Kim M, Kim J, Hwang J (2015). Development and performance test of a ZnO nanowire charger for measurements of nano-aerosol particles. J Sens Actuators A.

[22] Liu Z, Zhu R (2010). Electric-field-assisted growth and alignment of ZnO nanowires in device fabrication. J Phys D Appl Phys.

[23] Zong X, Zhu R (2014). An electric-field assisted growth control methodology for integrating ZnO nanorods with microstructures. Nanoscale.

[24] Yang WM, Zhu R, Zong XL. A MEMS based discharger using nanowires. Proceedings of the 13th IEEE International Conference on Nanotechnology. 5–8 Aug 2013. p. 360–5.

[25] Loeb LB (1965). Electrical coronas, their basic physical mechanisms.

[26] Raizer YP (1991). Gas discharge physics.

[27] Forbes RG, Edgcombe CJ, Valdre U (2003). Some comments on models for field enhancement. Ultramicroscopy.

[28] Peek FW (1929). Dielectric phenomena in high voltage engineering.

[29] Adachi M, Kousaka Y, Okuyama K (1985). Unipolar and bipolar diffusion charging of ultrafine aerosol particles. J Aerosol Sci.

